# A Women-Centred Approach: Development of a Randomized Controlled Trial Evaluating the efficacy of a Human-Supported Online Intervention for Depression and the Metabolic Signature of Personalized Treatment Response

**DOI:** 10.1192/j.eurpsy.2026.12049

**Published:** 2026-07-31

**Authors:** N. Angarita Osorio, N. Núñez Pazmiño, A. García Estela, L. Terraneo, O. Pozo, F. Colom

**Affiliations:** 1 Hospital del Mar Research Institute, Barcelona, Spain; 2 Università degli studi dell’insubria, Varese, Italy

## Abstract

**Introduction:**

Depressive disorders disproportionately affect women, who experience distinct biological and sociocultural risk factors that contribute to differential symptom profiles and disparities in treatment access and outcomes. Current interventions often lack gender sensitivity and personalization.

**Objectives:**

This study aims to evaluate the efficacy of the 3D Program, a human-supported, internet-based cognitive behavioral therapy (iCBT) intervention tailored for women with mild to moderate depressive symptoms, and to explore metabolic biomarkers associated with treatment response.

**Methods:**

In a single-blind, randomized controlled trial, 120 women aged 18 to 45 years will be allocated to either the 10-week 3D Program or psychoeducational video condition. Both groups will continue treatment as usual. The 3D Program combines personalized therapist support, self-guided digital modules, and a moderated online peer-support group, with materials co-designed by women with lived experience of depressive symptoms. The primary outcome is the change in depressive symptom severity, measured by the 17-item Hamilton Depression Rating Scale at 12- and 26-weeks post-baseline. Secondary outcomes include functioning, quality of life, perceived stress, and menstruation-related distress. Additionally, biological samples (plasma, saliva, urine, hair, and fingernails) will be analyzed to assess metabolic markers of tryptophan metabolism and adrenal steroid pathways. Data will be analyzed using linear mixed-effects models and multivariate techniques to examine treatment effects and biomarker associations. The trial is registered at ClinicalTrials.gov (NCT07060690) and received ethical approval from the Drug Research Ethical Committee of the Hospital del Mar Research Institute (2023/11236/I).

**Results:**

The intervention is hypothesized to reduce depressive symptoms more effectively than psychoeducation alone. Analysis of metabolic markers may further help identify biological correlates of treatment response, contributing to the development of personalized interventions.

**Conclusions:**

This trial seeks to advance gender-responsive and biomarker-informed mental health care. If effective, the 3D Program could offer a scalable, accessible adjunct to standard care in public health systems, improving outcomes for women with depressive symptoms and informing future precision-psychiatry approaches.

**Disclosure of Interest:**

None Declared

